# Road Traffic Noise and Diabetes: Long-Term Exposure May Increase Disease Risk

**DOI:** 10.1289/ehp.121-a60

**Published:** 2013-02-01

**Authors:** Wendee Nicole

**Affiliations:** Wendee Nicole, based in Houston, TX, has written for *Nature*, *Scientific American*, *National Wildlife*, and other magazines.

Noise is an environmental stressor that stimulates the body’s sympathetic nervous system and the hypothalamus–pituitary–adrenal axis, leading to increased blood pressure, heart rate, and levels of the “stress hormone” cortisol. Past research has associated exposure to traffic noise with cardiovascular disease, and the mechanisms of action hypothesized to underlie this association suggest that noise may also increase diabetes risk. Investigators now report that long-term exposure to residential road traffic noise was, in fact, associated with increased diabetes incidence in a Danish cohort [*EHP* 121(2):217–222; Sørensen et al.].

Glucocorticoid hormones, a group that includes cortisol, inhibit insulin secretion and reduce sensitivity to insulin by the liver, muscle, and fat tissue. Studies have linked sleep disturbances to low morning glucose levels, reduced insulin sensitivity, and changes in appetite regulation.

The investigators used data previously collected in the Danish Diet, Cancer and Health cohort study, which involved 57,053 individuals aged 50–64 at enrollment who were followed for an average of 9.6 years. For the present study, the team excluded any individuals who had developed cancer or diabetes prior to enrollment. Of the 50,187 eligible participants, there were 3,869 cases of incident diabetes available for the analysis. The investigators did not distinguish between type 1 and type 2 diabetes. However, since type 1 diabetes typically develops in childhood, most of the cases in the present study were likely type 2. In a second analysis, they excluded diabetes cases that had been identified solely on the basis of a history of undergoing blood glucose tests, creating a stricter definition for diabetes incidence.

**Figure:**
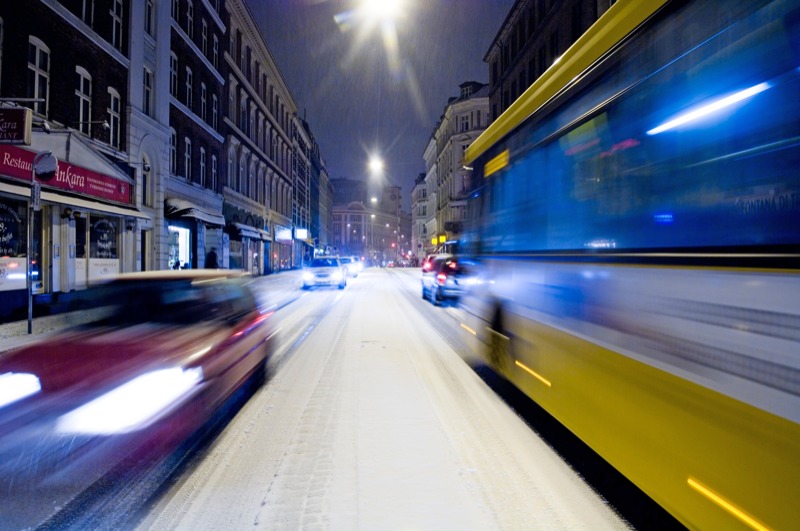
Vesterbrogade Street, Copenhagen, Denmark. Findings from a Danish cohort suggest exposure to road traffic noise may put residents at risk for diabetes. © Rune Johansen/Getty Images

Exposure to road traffic noise was estimated for each participant’s present and historical addresses from 1988 through 2006. The investigators used SoundPLAN, a model that incorporates data from a Danish national road and traffic database on number of vehicles, distribution of heavy and light vehicles, road type, and building characteristics. For each address they also estimated railway and airport noise as well as traffic pollution, which has been associated with diabetes in other studies.

Participants with diabetes were more likely to be men than women, had a higher body mass index and waist circumference, were older at enrollment in the study, smoked more or were exposed to more tobacco smoke, had lower education levels, ate less fruit and vegetables, engaged in less physical activity, and—as hypothesized—were exposed to higher levels of road traffic noise compared with the cohort as a whole.

Each 10-decibel increase in average road traffic noise at the current residence was associated with a statistically significant 8% increased risk of incident diabetes, increasing to 11% when road traffic noise was estimated for all the places an individual had lived in the previous 5 years. When the investigators applied the stricter definition of diabetes, they found an 11% increase in risk per 10-decibel increase in road traffic noise at the current residence, increasing to 14% when based on the previous 5 years. These associations held after adjusting for traffic pollution. There was no association between railway noise and diabetes.

The investigators conclude that reducing urban noise could improve population health, and specifically reduce the risk of diabetes. Future studies should seek to confirm the relationship between noise exposure and diabetes, and determine how traffic noise and air pollution interact to influence the risk of this disease.

